# Supplementary material for: Cross-national study of communal attitudes toward individuals with intellectual disabilities in sub-Saharan Africa: Cameroon vs. Ghana
Source: PLoS One. 2021 Sep 28;16(9):e0257482. doi: 10.1371/journal.pone.0257482 (PMC8478177; doi:10.1371/journal.pone.0257482)
Supplement: Attachment — Submitted filename: Response_to_comments.docx [file pone.0257482.s001.docx]

Dear Editor,

Thanks for considering our paper for publication and do appreciate the feedback from the two reviewers. We have considered the suggestions made by the two reviewers and incorporated in our revised manuscript. The changes made have been highlighted in yellow. Below is our point-by-point response to the reviewers’ comments.

| Comments | Response | Page number |
| --- | --- | --- |
| Reviewer 1 |  |  |
| There has been some research about the CLAS-ID being mainly useful for university students, who it was developed for, and not lay people or health professionals. Maybe comment on the applicability of the study results to the general population and/or government stakeholders. | Thanks for this comment. We have stated the participants were from diverse backgrounds and probably reflects the diversity in the society. In this sense, the findings could be taken in consideration in effort towards promoting the well-being of persons with disabilities. | See pages 8,27 |
| One limitation of the CLAS-ID is that there are conflicting findings about the purpose and value (positive or negative) of the sheltering subscale. Please comment: Ouellette-Kuntz et al. 2003 and Ouellette-Kuntz et al. 2012 comment on this, I believe. | Thanks for this comment. Yes, Ouellette-Kuntz et al. 2003 indicated that as a limitation which has been reference in our revised paper. However, we have added further that there are limited facilities for individuals with ID in the study environments. In view of this, the study participants cannot be influenced by their profession or programme of study. | See pages 4-5 |
| In the introduction, provide stronger rationale for looking at differences by gender, as that was a key study hypothesis. | We have indicated the need for studying the association between gender and attitudes towards individuals with ID. In particular, the onset of a child with disability usually leads to conflict at home which sometimes leaves the female parent as a caregiver. It is against this background that we came up with our third hypothesis. | See page 3 |
| In the introduction, provide stronger rationale for the Theory of Planned Behaviour, and comment on its other constructs (subjective norms, perceived behavioural control) or omit. | We have decided to omit this section from the revised paper. | See page 4 |
| What program or discipline were the students in? Different groups of students (e.g. law vs. social care students) have been shown to have different attitudes. | Thank you for your comment. The universities have their primary focus which became variable for comparison. Comparing based on individual programs could have led to comparison of over 10 specializations which might not be interesting to report. | See page 9 |
| Comment on whether gathering data from students at the end of lecture could have influenced participation, and whether participation in the study was voluntary or for course credit. | The data collection process did not influence participation. That’s why the authors stood behind and those who wanted to participated in the study did so willingly. Enough time was also given to the participants to return the completed questionnaire. | See page 11 |
|  |  |  |
| Reviewer 2 |  |  |
| I would like to thank the authors for exploring an interesting and important topic. The study explores the attitudes towards people with intellectual disability in the geographical context of Cameroon and Ghana while investigating psychometric properties of a questionnaire--the community living attitude scale for intellectual disability (CLAS-ID)--measuring the attitudes towards people with intellectual disability (CLAS-ID). The study recruits 741 university students from two public universities in Cameroon and Ghana, selected based on convenience. The study concluded the CLAS-ID is a sound instrument to measure attitudes towards people with ID in Cameroon and Ghana contexts, while attitudes towards people with intellectual disability among participants were found to be ambivalent. Additional findings were also reported in the manuscript. The authors provided elaborate background information and justification for this study, and I agree that covering the topic is important to better understand the situation of people with ID, especially in low and middle-income countries context, and to endorse the inclusion of the said people in society. | Thanks for your kind words. |  |
| This study uses both CFA and PCA to explore the CLAS-ID underlying factors, however, both analyses yielded different results. The authors might want to discuss further the rationale of using both analyses in this study, and/or how the reader should interpret the results. | Thanks for this. We have provided reference to the reason for conducting both analyses. We wanted to be as transparent as possible and as such, the computation of CFA and PCA. We wanted readers to know how the tool fared on both. However, we have directed future researchers to focus on the PCA which supported the structural validity of the tool. | See page 12 |
| Page 11 [With respect to the Cameroonian data, the questionnaires were both in English and French since both languages are the medium of instruction and officially recognized languages]  Does this mean the Cameroonian participants receive two versions of the CLAS-ID, English and French? If yes, do the two versions are considered identical instruments? Please clarify. | Thanks for this. The English and French versions were on the same document given to the students. Each statement was in both French and English. | See page 11 |
| Under the 'Predictors of Attitudes' subheading, there is a discussion about the association between the CLAS-ID sub-scales, which does not seems relevant with the sub-headings topic. Please clarify. [page 19] 'the association between the sub-scales…. | Thanks for this. We indicated in the method that before conducting regression, we first computed the relationship between the sub-scales. This is the reason why the results have been presented in this order. | See page 13, 19 |
| [page 2] From the perspectives of parents and individuals with IDs, empirical evidence suggests that they continue to suffer discrimination and rejection by other people in sub-Saharan African societies. The authors might want to elaborate more on the examples of discrimination and rejection of people with ID in sub-Saharan societies. I think the information could help the readers to better understand the situation of people with ID in Ghana and Cameroon context. | Thanks for this. We have expanded and given examples of the discrimination and rejection suffered by parents. | See page 2 |
| page 10] 'almost all the academics responded that the content was appropriate for data collection  Does this mean some academics disagree that the content of the instrument appropriate for data collection? If yes, then the author might want to provide more discussion about this. | Thanks for this. We wanted to state that all the participants provided feedback which was incorporated in the draft used for data collection. | See page 10 |
| The Author might want to add a reference for this statement:  [page 2] The onset of an ID is usually diagnosed before 18 years of age, with leading causes attributed to genetic factors and neurodevelopmental, neurological, and other medical conditions. | Thanks for this. Reference added. | See page 2 |
| The finding regarding participants' ambivalent attitude towards people with ID, is not included in the abstract. The authors might want to consider adding the information in the abstract. | The findings on the ‘ambivalence of participants on attitudes’ have been added to the reference. | See page 1 |

Thanks once and looking forward to a favourable feedback on our resubmission.
